# Supplementary figures and images for: Extracellular Vesicles and Cx43-Gap Junction Channels Are the Main Routes for Mitochondrial Transfer from Ultra-Purified Mesenchymal Stem Cells, RECs
Source: Int J Mol Sci. 2023 Jun 18;24(12):10294. doi: 10.3390/ijms241210294 (PMC10299354; doi:10.3390/ijms241210294)

# Restoration of mitochondrial function by REC-derived mitochondria in the non-contact system

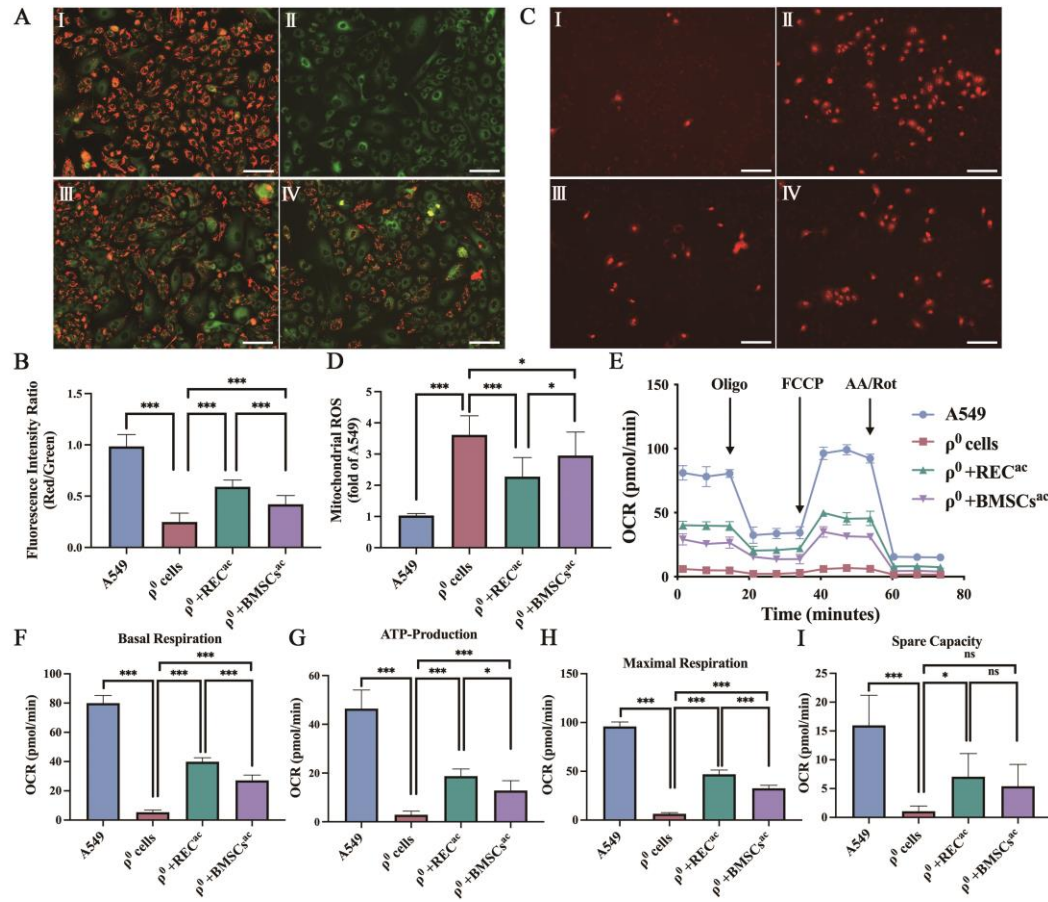

Supplement: Supplementary file 1 [file ijms-24-10294-s001.zip › ijms-2386049-supplementary.pdf]
